# Colonial transition as a major mediator of global health transition: lessons from the 2024 New Caledonia crisis

**DOI:** 10.7189/jogh.15.03004

**Published:** 2025-01-31

**Authors:** Pierre-Henri Moury, Marcelin Tromhae, Cécile Cazorla, Mathieu Série, Antoine Flahault, Emmanuel Couadau, Cynthia Fleury, Morgan Mangeas, Thierry De Greslan

**Affiliations:** 1 Université Grenoble Alpes-CHU Grenoble Alpes, Grenoble, France; 2 Service de réanimation, Centre Hospitalier Territorial Gaston-Bourret, Dumbea-Sur-Mer, New Caledonia, France; 3 Global Health Institute, University of Geneva, Switzerland; 4 Chaire ‘Humanité et Santé’, Conservatoire National des Arts et Métiers, GHU Paris Psychiatrie et Neurosciences, Paris, France; 5 UMR ENTROPIE 9220, Institut de Recherche pour le Développement. IRD, CNRS, UR, New Caledonia, France; New Caledonia is currently seeking its future institutional framework through violent, politically-driven clashes within the population. The insurrections in May 2024, fuelled by political, and social divisions, also targeted the healthcare system in an unprecedented manner. Here we summarise the situation from a global health perspective and outline its link with colonial transitions. The territory now faces new health-related challenges because of trends in ageing (due to demographic transitions) and the transition from infectious to noncommunicable diseases (NCDs), especially due to disparities between ethnic groups. Research has shown the Melanesian (Kanak) and Oceanian populations had a higher average body mass index than European (*P* < 0.001). Simultaneously, the incidence of leptospirosis (which is associated with rainfall) in 2021–22 were higher in the communal district where the Kanak group formed the majority of the population, compared to the western and southern coasts, where European people lived. Among the NCDs, issues with mental health predominate the literature, especially among the young, males, and the Kanak population. We therefore call for a deep consideration of this problem, considering all the cases that are emerging due to the new civil unrest that targeted all the society. Simultaneously, the 2020 and 2021 referendums for independence during the COVID-19 pandemic lockdowns revealed the deep-seated impacts of global health transition embedded within the colonial trauma, as disease control and its consequences were an essential prerequisite for the political debate. These vulnerabilities highlighted the urgency for targeted interventions, necessitating an approach adapted to community care that respects the New Caledonia people's cultural practices. We also call for a field-based experimental approach that must emphasise sustainable health, inequality reduction, the One Health approach, and climate change. Ultimately, integrating sociological insights into health policies is crucial for restoring dignity, addressing trauma, and preparing for future crises, fostering a more inclusive and resilient society.

New Caledonia is currently seeking its future institutional framework through political battles and violent clashes within the population. Numerous injuries have been reported and healthcare facilities destroyed [1,2]. This unprecedent destruction of the region’s health system, unprecedented in the context of the modern French Republic, will negatively impact the burden of many diseases in a near future. Here we aimed to outline the background of these struggles and show how colonial transitions are intertwined with global health transitions, which themselves are often described through three main lenses: epidemiological, demographic, and climatic. Epidemiological transitions are defined by a shift from a heavy burden of infectious diseases to a major prevalence of noncommunicable diseases (NCDs), which currently account for about 80% of all deaths worldwide, and are thus closely connected to the ageing of the populations – *i.e.* the demographic transition [3]. Infectious disease, meanwhile, remain deeply dependant on climate changes and the related surges of zoonotic risks [4].

Here we provide a historical background within the global health transitions by presenting the key figures of the epidemiology of New Caledonia, before discussing the impact of the current crises. In doing so, we advocate for the implementation of a global health strategy that considers colonial transition as a significant mediator. We then emphasise the potential of care and its components – thoughtfulness, responsibility, compassion, attention to the needs of others – as a leverage tool for the restoration of dignity [5].

## NEW CALEDONIA: A MULTICULTURAL SOCIETY

New Caledonia is an autonomous territory of the French Republic located in the South Pacific. It has a multicultural population composed of Melanesian indigenous people (known as Kanak), Polynesians, Asians, and Europeans. According to its self-declared 2019 census which collected data on approximately 270 000 inhabitants [6], its population comprised about 40% Kanak, 30% Europeans, and 8% Polynesians from Wallis-and-Futuna 8%, while another 8% declared themselves as being of different origin, 7% claimed more than one origin, and 8% did not declare themselves as belonging to any group.

All these groups perceive the major periods of human history differently; however, such divergences also exist within their communities [7]. Although the educational system based on the French model was likely a factor in this diversification, behavioural responses to the learning scheme were mainly dependent on the ethnic group [8]. This is further confirmed by the fact that ethnic identity was found to be a strong mediator of self-esteem, with adolescents of European origin exhibiting it significantly higher than others [9]. Such social inequities persist despite education and were possibly among the main reasons behind the recent civil unrest (**Table 1**).

**Table 1:** Characteristics of each ethnic group by specific professions, educational levels, and health status

	Kanak	Europeans	Wallis-et-Futuna	More than one origin	Polynesian	*P*-value*
**Profession, n (%)†**						
Farmers	2375 (81)	355 (12)	43 (1.5)	126 (4)	19 (1)	
Executives positions	1441 (14)	7530 (73)	276 (3)	969 (9)	154 (1.5)	
Intermediate Professions	5473 (26)	10922 (52)	1531 (7)	2748 (13)	463 (2)	
Employees	13 017 (49)	6526 (24)	3224 (12)	3651 (13)	816 (3)	
**Education, n %†**						
No diploma	26 982 (66)	4141 (10)	5744 (14)	3169 (8)	1188 (3)	
Master’s degree or higher	2790 (13)	16 550 (75)	530 (2)	2013 (9)	255 (1)	
**Health**‡	n = 2205	n = 521	n = 113	n = 439	n = 27	
Age in years, x̄ (95% CI)	42 (32–52)	47 (37–56)	42 (31–50)	40 (30–51)	47 (32–53)	<0.001
Diabetic, n (%)	78 (16)	9 (7.6)	9 (24)	10 (0)	3 (27)	0.009
Non-diabetic, n (%)	405 (84)	109 (92)	28 (76)	99 (100)	8 (73)	
Height in cm, x̄ (95% CI)	164 (159–171)	169 (163–176)	170 (164–177)	166 (161–173)	174 (167–177)	<0.001
Weight in kg, x̄ (95% CI)	80 (69–93)	74 (63–85)	100 (83–115)	79 (66–94)	100 (87–115)	<0.001
Body mass index in kg/m^2^, x̄ (95% CI)	30 (25–34)	25 (22–29)	35 (30–39)	28 (24–34)	33 (29–39)	<0.001
Smoked tobacco, n (%)	153 (39)	1078 (64)	53 (55)	199 (57)	13 (62)	<0.001
Think about stopping alcohol last year, n (%)	94 (21)	450 (31)	15 (19)	75 (21)	5 (25)	<0.001
Smoked cannabis every day, n (%)	15 (27)	144 (39)	0 (0)	28 (42)	1 (25)	0.3

CI – confidence interval, x̄ – mean

*Fisher’s exact test or Kruskal-Wallis test.

†Data from the 2019 census [10].

‡Data from the 2021–22 Baromètre steps survey of 2880 inhabitants presented for adults [11].

## HISTORICAL BACKGROUND

**Figure 1:**
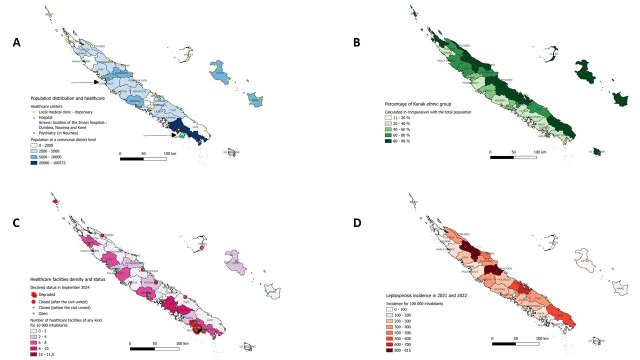
Maps of New Caledonia: demographic, social, and health disparities in the context of the 2024 civil unrest. **Panel A.** Number of inhabitants for each communal district of New Caledonia and the different healthcare centres, according to the 2019 census. **Panel B.** Percentage of Kanak (Melanesians) inhabitants in each communal district. The representation of the Kanak people was higher in the eastern part of the territory and the Islands province. **Panel C.** Number of healthcare facilities of any kind for 10 000 inhabitants and their declared status in September 2024 after the civil unrest. The reported status was either closed because of the riots, closed before the crisis because of the lack of human resources, or open (unknown cases were not reported). **Panel D.** Burden of leptospirosis in New Caledonia in each communal district according to the mean incidence of 2021 and 2022.

The Kanak had been the primary inhabitants of the islands; from the 19th century onwards, there were several waves of settlement by groups of Europeans, Polynesians, and Asians. Akin to Australia, many Europeans arrived during the second half of the 19th century, when New Caledonia became a penitentiary colony. Religion, trade, mining, and the geostrategic position within the South Pacific were key drivers of settlement. These groups settled mostly on the western part of the main island, which is still home to most of the population; the native population mostly remained in its northern and eastern parts (**Figure 1**). From an anthropological perspective, this change introduced new ontologies, best understood as the various definitions of the boundaries between oneself and others [12], into the millennia-old Melanesian culture. One of the most intriguing aspects of Kanak ontology is its deep-seated continuity between humans and nature, in which the concept of “others” includes non-human entities and the ancient [12]. For centuries, life was regarded as the breath transmitted through the interaction of nature, the ancient, and the newborn.

### Decolonisation process

As we mentioned, the turn of the 19th century saw the disruption of the ancient ways. From 1853 (the founding year of the French colony) until today, the natives struggled for dignity, as did the displaced European population and the labourers from Asia. The contact with the new settlers also introduced new infectious diseases and, according to some authors, a drop of at least 50% in the Kanak population [13]. Political tensions peaked in the 1980s, culminating in the Matignon Accords that recognised a decolonisation process. The process was reinforced by the Nouméa Accord in 1998, which aimed to address social and economic disparities through the transfer of governance across various sectors: economy, mining, transport, environment, and health. This treaty also included a provision outlining three referendums to decide whether New Caledonia should become fully independent. They took place in 2018, 2020, and 2021, with the latter two occurring during the COVID-19 pandemic. The process was intended to foster the creation of local inclusive citizenship and a shared future [7]. Despite impressive achievements, however, the referendums have polarised the population through political debates and social divisions. The prospect of granting political rights to inhabitants with a ten-year residence in the territory or country fuelled much of the anger. However, some underlying reasons were also related to the consequences of the COVID-19 pandemic.

### COVID-19 pandemic: an overlap with the decolonisation process

New Caledonia successfully implemented an early elimination strategy through two lockdowns [14–16], established mainly for epidemiological reasons, but also because most of the community leaders called for strong isolation measures due to their existing memories of the introduction of infectious diseases. The second referendum took place during a zero-COVID-19 period in 2020; 86% of the population took part, with 53% voting against and 47% for independence. Social and political tensions emerged, but did not lead to major violence. The independentists asked for the third referendum after the second lockdown, which had been established due to the circulation of the alpha variant in April 2021. At that time, barely 10% of the population was vaccinated [16]. The introduction of the delta variant across the border in September 2021 led to a surge in cases, necessitating a third lockdown and an urgent adaptation of the health system in which less than 30% of the population being vaccinated [14,17]. The toll was dramatic – there were almost 100 deaths per 100 000 population in three months [17].

In accordance with their Melanesian culture, community leaders called for a one-year mourning period and proposed to delay the third referendum, which had been scheduled for December 2021 [18]. Yet while COVID-19 incidence had decreased to a manageable level at the time of the vote, a decision to boycott the referendum was made. The participation dropped to 44%, with 96.5% voting against independence and 3.5% in favour. Here, we refrain from delving into the political motivations behind the decision to hold or boycott the referendum amidst the pandemic. As psychiatrist Dr Frantz Fanon taught us in the 1950s, colonisation may lead to alienation, shaping a path from psychological difficulties to psychiatric disorders [19].

## GLOBAL HEALTH TRANSITIONS

Our goal here is to accurately map the health inequities related to these events (**Figure 1**). A recent survey found that two-thirds of the population was affected by obesity or overweight, with a significantly higher body mass index for the Oceanian population and teenagers [20–22] (**Table 1**).

In view of NCDs, mental health disorders emerge as a significant concern during the colonisation transition, in line with the mechanisms discussed by Fanon [23]. These included distress due to the region’s political situation and other circumstances prevalent in the context of the Pacific Islands, which have one of the highest rate in suicide worldwide. Studies conducted in New Caledonia reported specific alcohol and drug abuse challenges, as well as high rates of suicide for young Kanak males [23–25] (**Table 1**). The archipelago also remains vulnerable to climate change and tropical diseases (**Figure 1**, Panel D), with a notable association between outbreaks of leptospirosis, a neglected tropical bacterial zoonotic disease, and heavy rain influenced by ocean stream patterns [26] – a challenge exacerbated by issues with safe water supplies. The distribution of cases is the highest in areas where the majority of Kanak people reside, yet simultaneously, healthcare facilities are primarily concentrated in the South and along the Western coast.

## THE 2024 CIVIL UNREST

At the time of writing this manuscript, we were unable to predict the extent of the consequences of the civil unrest. Thirteen deaths from violent causes have been reported, with the number of wounded or those individuals whose treatment had been delayed being challenging to ascertain. The Greater Nouméa area, encompassing the capital of Nouméaand the surrounding cities, saw most of the violence. Pharmacies, dialysis centres, and general practitioner offices were burned or destroyed as collateral, leading to an unprecedented emigration of healthcare workers (**Figure 1****,** Panel C; Figure S1 in the **Online Supplementary Document**) [1,2]. Road barricades prevented entry into the hospital, necessitating the establishment of a temporary pontoon in the mangrove to allow the healthcare workers access (Figure S2 in the **Online Supplementary Document**).

The psychological trauma stemming from the violence and economic losses is expected to have long-lasting consequences, with victims among all classes of New Caledonia – a context where human resources for basic healthcare are already low. We therefore underscore the importance of ensuring proper public health conditions as an essential prerequisite for facilitating democratic debates.

## GLOBAL HEALTHCARE STRATEGY

We advocate for the consideration of the psychological impact of the violence, economic loss, and the colonial transition through a differential approach in community-targeted care. This would begin with a mapping of vulnerabilities specific to the context of New Caledonia, where determinants of mental health are currently poorly studied, and where the mechanism behind the power imbalance and the relation between aggressors and victims have become more fluid since the May 2024 crisis. To address the current epistemological issue, we suggest the prioritisation of a field-based experimental approach [27]; multi-faceted interventions should be implemented, tested, and evaluated to address trauma and mental health initiatives dedicated to young people with a focus on restoring dignity to the many affected individuals [28] (Figures S3 in the **Online Supplementary Document**). These reflections were part of the ‘Do Kamo road map’, developed through a rigorous participatory method aimed at accelerating the transition towards an equitable, sustainable, and local health system [29]. However, at the time of writing, emergencies, economic burdens, and crises have reversed any progress made in this project.

## CONCLUSION

Being ‘inside’ and ‘outside’ of France at the same time has shaped New Caledonia, acting a double burden. Colonisation has been shaping the sociology, landscape, and health of the territory – from mental health issues to demography and infectious diseases – for nearly 200 years. A change in paradigm can only come from a change in the approach to these issues by including the local populations as primary actors. Addressing these decolonisation issues within the French Republic is not a paradox; it aims at ensuring greater democratic, cultural, and knowledge diversity within the structures of global health. This approach should take precedence to mitigate the potential exacerbation of the current situation.

## Additional material


Online Supplementary Document

## References

[1] Riahi S, Detcheverry G, Favenecc C. Crise en Nouvelle-Calédonie: les départs de médecins libéraux font craindre des déserts médicaux. franceinfo. 30 July 2024. Available: https://la1ere.francetvinfo.fr/nouvellecaledonie/crise-en-nouvelle-caledonie-les-departs-de-medecins-font-craindre-des-deserts-medicaux-1508744.html. Accessed: 8 October 2024.

[2] Baroux N Maire L Cadic L Lemaitre A-F Borceux P Glasman B Riots in New Caledonia: Impact of constrained management on peritoneal dialysis patients. Bull Dial À Domic. 2024;7:89–99. 10.25796/bdd.v7i3.84663

[3] Abegunde DO Mathers CD Adam T Ortegon M Strong K The burden and costs of chronic diseases in low-income and middle-income countries. Lancet. 2007;370:1929-38. 10.1016/S0140-6736(07)61696-1 18063029

[4] Baker RE Mahmud AS Miller IF Rajeev M Rasambainarivo F Rice BL Infectious disease in an era of global change. Nat Rev Microbiol. 2022;20:193–205. 10.1038/s41579-021-00639-z 34646006 PMC8513385

[5] Tronto JC. Un monde vulnérable: Pour une politique du care. Paris, France: La Découverte; 2009.

[6] Institut de la Statistique et des Études Économiques-Nouvelle-Calédonie. Une mosaïque pluriethnique. Available: https://www.isee.nc/population/recensement/communautes. Accessed: 24 May 2024.

[7] Sand C Bolé J Ouetcho A-J Les aléas de la construction identitaire multi-ethnique en Nouvelle-Calédonie: quel passé pour un avenir commun? J Soc Océan. 2003117:147–69. 10.4000/jso.1252

[8] Frayon S Swami V Wattelez G Nedjar-Guerre A Galy O An examination of procrastination in a multi-ethnic population of adolescents from New Caledonia. BMC Psychol. 2023;11:1. 10.1186/s40359-022-01032-y 36593477 PMC9806450

[9] Frayon S Swami V Wattelez G Todd J Galy O Associations between weight status, body satisfaction, ethnic identity and self-esteem in Oceanian adolescents. Pediatr Obes. 2021;16:e12824. 10.1111/ijpo.12824 34184838

[10] Institut de la Statistique et des Études Économiques-Nouvelle-Calédonie. Recensement. Available: https://www.isee.nc/population/recensement/. Accessed: 24 May 2024.

[11] Gouvernement de la Nouvelle-Calédonie. Adult Health Barometer 2021-2022 (BSA21). Available: https://data.gouv.nc/explore/dataset/data_barometre_sante_adulte_2021_2022/.

[12] Descola P. Par-delà nature et culture. Gallimard. Paris, France: Gallimard; 2005.

[13] Sand C. Hécatombe océanienne. Histoire de la dépopulation du Pacifique et ses conséquences (XVIe-XXe siècle). Tahiti, French Polynesia: Au Vent des Iles; 2023.

[14] Kerbaj J Cazorla C De Greslan T Serie M Gourinat AC Marot B COVID-19: The New Caledonia Experience. Clin Infect Dis. 2020;71:2279–81. 10.1093/cid/ciaa600 32415955 PMC7239214

[15] Moury P-H Ochida N Motiejunaite J Collart V Série M Gervolino S Impact of lockdown on cardiovascular disease hospitalizations in a Zero-COVID-19 country. Public Health. 2023;217:98–104. 10.1016/j.puhe.2023.01.029 36867989 PMC9894760

[16] Moury P-H Gourinat A-C Riou O Laumond S Dupont-Rouzeyrol M Cazorla C Successful COVID-19 elimination after an alpha variant outbreak in a “safe travel zone”. Travel Med Infect Dis. 2021;44:102202. 10.1016/j.tmaid.2021.102202 34767909 PMC8577871

[17] Ochida N Dupont-Rouzeyrol M Moury P-H Demaneuf T Gourinat A-C Mabon S Evaluating the strategies to control SARS-CoV-2 Delta variant spread in New Caledonia, a zero-COVID country until September 2021. IJID Reg. 2023;8:64-70. 10.1016/j.ijregi.2023.06.004 37583482 PMC10423666

[18] Whaap B, Madec A. Le sénat coutumier décrète un “deuil kanak” d’une année et se positionne en faveur du report du référendum. franceinfo. 9 November 2021. Available: https://la1ere.francetvinfo.fr/nouvellecaledonie/le-senat-coutumier-decrete-un-deuil-kanak-d-une-annee-et-se-positionne-en-faveur-du-report-du-referendum-1149691.html. Accessed: 9 October 2024.

[19] Fanon F Peau noire, masques blancs. Socio-anthropologie. 2018;37:189–93. 10.4000/socio-anthropologie.3340

[20] Frayon S Wattelez G Paufique E Nedjar-Guerre A Serra-Mallol C Galy O Overweight in the pluri-ethnic adolescent population of New Caledonia: Dietary patterns, sleep duration and screen time. Lancet Reg Health West Pac. 2020;2:100025. 10.1016/j.lanwpc.2020.100025 34327376 PMC8315340

[21] Agence Sanitaire et Sociale de Nouvelle-Calédonie. Epidémiologie. 2024. Available: https://www.santepourtous.nc/les-thematiques/mange-mieux-bouge-plus/obesite-en-nc/epidemiologie. Accessed: 9 October 2024.

[22] Agence Sanitaire et Sociale de Nouvelle-Calédonie. Enquête Steps Baromètre Santé Adulte 2021-2022. Nouméa, New Caledionia: Agence Sanitaire et Sociale de Nouvelle-Calédonie; 2023. Available: https://www.santepourtous.nc/images/st-barometre-sante-adulte/st-pdf/st-rapport-baromtre-v10-lq.pdf. Accessed: 9 October 2024.

[23] Mathieu S de Leo D Koo YW Leske S Goodfellow B Kõlves K Suicide and suicide attempts in the Pacific Islands: A Systematic Literature Review. Lancet Reg Health West Pac. 2021;17:100283. 10.1016/j.lanwpc.2021.100283 34734201 PMC8495100

[24] Goodfellow B Kõlves K Selefen A-C Massain T Amadéo S De Leo D The WHO/START study in New Caledonia: A psychological autopsy case series. J Affect Disord. 2020;262:366–72. 10.1016/j.jad.2019.11.020 31740112

[25] Vignier N Lert F Salomon C Hamelin C Kava drinking associated with suicidal behaviour among young Kanaks using kava in New Caledonia. Aust N Z J Public Health. 2011;35:427–33. 10.1111/j.1753-6405.2011.00737.x 21973249

[26] Weinberger D Baroux N Grangeon J-P Ko AI Goarant C El Niño Southern Oscillation and Leptospirosis Outbreaks in New Caledonia. PLoS Negl Trop Dis. 2014;8:e2798. 10.1371/journal.pntd.0002798 24743322 PMC3990495

[27] Fleury C Tourette-Turgis C Mukwege D Lauffenburger M Un essai d’approche philosophique clinique des terrains à l’Hôpital de Panzi en République démocratique du Congo. Rech Éducations. 2025;28–29:12oj2.

[28] Fleury C. La Clinique de la dignité. Paris, France: Editions Seuil; 2023.

[29] Gouvernement de la Nouvelle-Calédonie. Do Kamo être épanoui – Plan de santé calédonien. Nouméa, New Caledionia: Gouvernement de la Nouvelle-Calédonie; 2018. Available: https://gouv.nc/sites/default/files/atoms/files/brochure_do_kamo_etre_epanoui_0.pdf. Accessed: 8 October 2024.

