# Supplementary material for: Colonial transition as a major mediator of global health transition: lessons from the 2024 New Caledonia crisis
Source: J Glob Health. 2025 Jan 31;15:03004. doi: 10.7189/jogh.15.03004 (PMC11781806; doi:10.7189/jogh.15.03004)
Supplement: Online Supplementary Document [file jogh-15-03004-s001.pdf]

Supplement to: Moury PH, Thromae M, Cazorla C, Série M, Flahault A, Couadau E, Fleury C, Mangeas M, De Greslan T. Colonial transition as a major mediator of global health transition: lessons from the 2024 New Caledonia crisis. J Glob Health. 2025;15:03004.

Figure S.1 description: consequences of the May 2024 civil unrest on the Greater Noumea Healthcare facilities

The Centre-Hospitalier-Territorial Gaston-Bourret is situated on the Dumbea communal district near the sea. A temporary pontoon was settled in the mangroove near the hospital to maintain an access for the healthcare workers

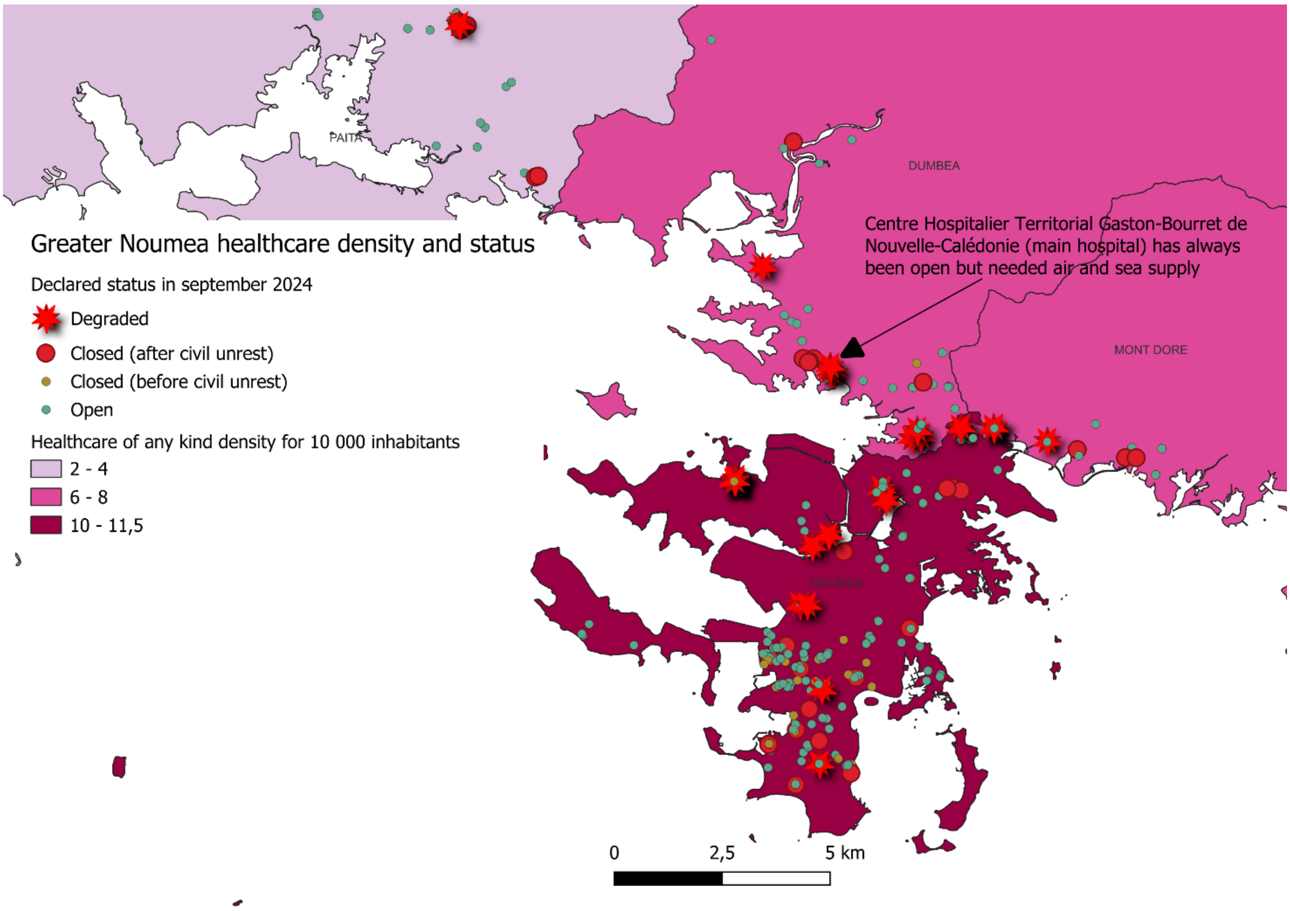

Figure S.2: picture from the temporary pontoon

Credit Dr Cécile Cazorla

The temporary pontoon was installed to keep an access for healthcare workers to the hospital.

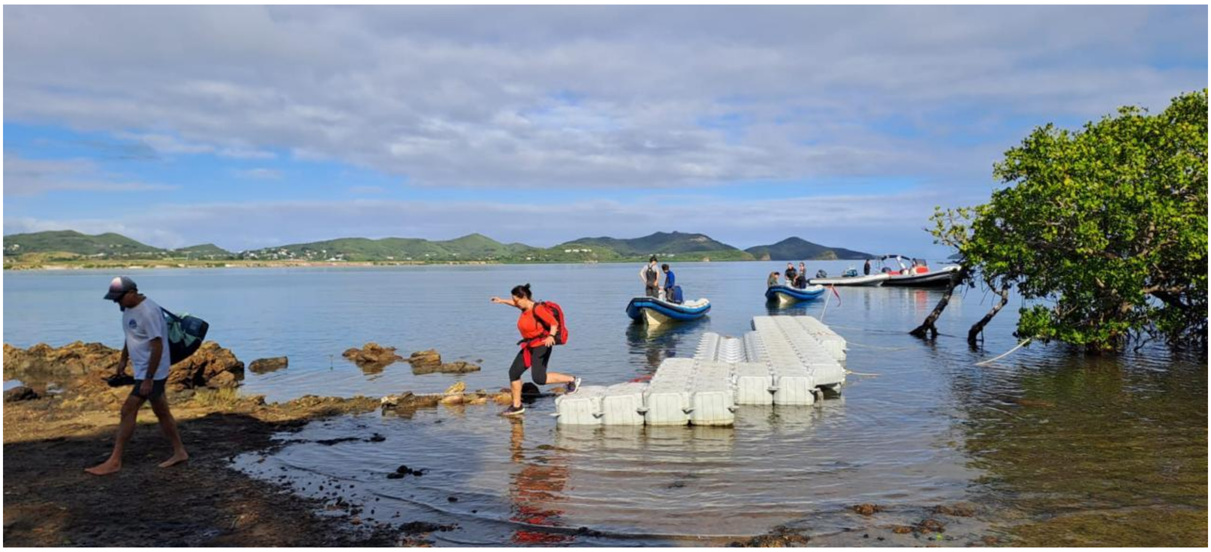

Supplementary materials 3: discussion on a health strategy for New Caledonia

Returning to the primary determinant of health in the Kanak population, the quality of interpersonal relationships within the community and environment are paramount, with an emphasis on seeking meaning behind illnesses deeply ingrained [1]. Policymakers must acknowledge the profound connection of the indigenous people of the archipelago when addressing healthcare needs in New-Caledonia. Therefore, we advocate for leveraging Care within the decolonization process as a tool for preparing for future crises, aligning with the French government's Global Health strategy (https://sante.gouv.fr/ministere/europe-et-international/la-sante-mondiale/),. This necessitates adequate resources. In the words of Nobel laureate Gabriel Garcia Marquez during his Stockholm lecture on the challenges of colonialism, “all creatures of that unbridled reality, we have had to ask but little of imagination, for our crucial problem has been a lack of conventional means to render our lives believable. This, my friends, is the crux of our solitude”.[2] With all of the five pillars of the French strategy in Global health we can implement concrete actions:

- (1) to contribute to sustainable health for all: mapping the vulnerabilities, reform the health insurances, accelerate the “Do Kamo road map”[3]. Promote bottom-up strategies.
- (2) to reduce health inequalities, working on all social and environmental aspects: adjust the health systems to the community levels by a scheme of local community healthcare workers, accelerate the numeric transition by building a territorial health data-hub, mental health issues must have a specific plan with a field-base experimental approach.
- (3) to strengthen the One Health approach to better prevent and prepare for future global health emergencies: maintain adequate water supply, zoonotic prevention by specific programs. Enhance public health methodology to bridge the gap to meet the criteria of 2030 WHO goals for everyone.
- (4) address the health consequences of climate change and the environmental impact of health systems: promote local knowledge and prevention targeting the NCDs and vulnerable populations such as mother-and-child care.
- (5) promote French and New Caledonia values, expertise, and research, innovation, and partnerships: respect the right for autonomy and community

empowerment. Focus on prevention and preparedness for the most vulnerable. Enhance the regional cooperation in South Pacific. Building a public health research and teaching institute bridging the institutions of New Caledonia such as Institut Pasteur, Institute de Recherche et Développement and the University of New Caledonia.

References

1 Pidjo A-T, Sabinot C, Hnawia E. Recherche de sens et stratégies de soins chez les Mwalebeng de Pouebo (Nouvelle-Calédonie). J Société Océan. 2023;7–20. doi:10.4000/jso.14994

2 Garcia Marquez G. The Nobel Prize in Literature lecture 1982. In: NobelPrize.org [Internet]. 1982 [cited 3 Jul 2021]. Available: https://www.nobelprize.org/prizes/literature/1982/marquez/lecture/

3 Do Kamo être épanoui - Plan de santé calédonien. Gouvernement de la Nouvelle-Calédonie; 2018. Available: https://gouv.nc/sites/default/files/atoms/files/brochure_do_kamo_etre_epanoui_0.pdf
